# Behavioural Evidence and Chemical Identification of a Female Sex Pheromone in *Anagrus atomus* (Hymenoptera: Mymaridae)

**DOI:** 10.1007/s10886-021-01272-z

**Published:** 2021-04-16

**Authors:** Penelope Zanolli, Desiderato Annoscia, Virginia Zanni, Francesco Nazzi, Francesco Pavan

**Affiliations:** Department of Agricultural, Food, Environmental and Animal Sciences, University of Udine, via delle Scienze 206, 33100 Udine, Italy

**Keywords:** Egg parasitoids, Eugenol, *Empoasca vitis*, GC-MS, Biological control

## Abstract

*Anagrus atomus* (L.) is an egg parasitoid involved in the biological control of *Empoasca vitis* (Göthe) in vineyards. Sex pheromones play a crucial role in mate finding for several parasitoid species and could be used for monitoring under field conditions. We carried out laboratory and field studies aimed at assessing the existence and identity of a possible *A. atomus* sex pheromone. We found that males were significantly attracted by virgin females independent of age. Males were not attracted to individuals of the same sex, but they were attracted by a crude extract from an unmated female and its polar fraction. Eugenol (4-allyl-2-methoxyphenol) was identified as the attractive substance and proved to be attractive not only in the olfactometer but also in another laboratory bioassay and under field conditions. Attraction of males, but not females, confirms that this is not an aggregation pheromone. This is the first sex-pheromone component identified in Mymaridae, however more compounds could be involved in the mating behaviour of *A. atomus*. The utility of a sex pheromone in *A. atomus* is discussed in the context of fitness returns.

## Introduction

Mate finding is a crucial step in the mating system of insects and the use of reliable information may increase both the probability of finding a mate and mating success. Several stimuli (e.g. visual and tactile) can be used for this purpose but chemicals produced by members of both sexes are mainly involved (Symonds et al. 2009). Depending on the function, the range of activity of pheromones can vary. Highly volatile compounds released by females are used by males for long-range orientation during mate finding (> 2 cm), whereas chemicals of relative low volatility mediate male courtship behaviour at close range (Steiner et al. 2006). Sex pheromones play a crucial role in mate finding in most hymenopteran parasitoids (Kainoh 1999; Ruther 2013). Until now, the sex-pheromone components of more than 20 hymenopteran parasitoid species, belonging to ten families, have been identified (Eller et al. 1984; Hrabar et al. 2015; Kainoh 1999; Keeling et al. 2004; Nichols Jr et al. 2010; Pompanon et al. 1997; Quicke 1997; Ruther 2013; Ruther et al. 2011; Salerno et al. 2012). In the family Mymaridae, evidence of a sex pheromone has been reported for one species only (Cormier et al. 1998); however, no sex-pheromone components have been identified so far. Parasitoids’ sex pheromones are more often produced by females, however there is increasing evidence of sex pheromone production by males as well (Steiner et al. 2006; Steiner and Ruther 2009; Ruther 2013).

Interest in sex pheromones for use in integrated insect pest management is usually limited to pheromones released by herbivorous pest insects (Mainers and Peri 2013; Witzgall et al. 2010). Among the semiochemicals associated with natural enemies, mostly kairomones and synomones are considered in pest control strategies (James 2003, 2005; James and Price 2004; Rodriguez-Saona et al. 2012). However, the possibility of capturing natural enemies with traps baited with live females (Jewett and Carpenter 2001) or with lures containing their sex pheromones (De Lury et al. 1999; Eller et al. 1984; Gabrýs et al. 1997; Itadani and Ueno 2014; Suckling et al. 2002) has been exploited for research purposes.

*Anagrus atomus* L. (Hymenoptera: Mymaridae) is a cosmopolitan egg parasitoid of leafhoppers (Arnò et al. 1988; Chiappini 1987; Huber 1986; Matteucig and Viggiani 2008; Triapitsyn 1998; Waloff and Jervis 1987) and plays an important role in the biological control of the green leafhopper *Empoasca vitis* (Göthe) (Hemiptera: Cicadellidae) in European vineyards (Böll and Hermann 2004; Böll and Schwappach 2003; Boller et al. 2004; Cerutti et al. 1991; Hermann and Eichler 2000; Pavan and Picotti 1993, 2009; Picotti and Pavan 1993; van Helden and Decante 2001; Vidano et al. 1988; Zanolli and Pavan 2011, 2013). *Anagrus atomus* was suspected to be a complex of species, specialized on different leafhopper species, distinguishable on the basis of morphological characters, cuticular hydrocarbons and genetic analysis (Chiappini 1987; Chiappini et al. 1996; de León et al. 2009; Floreani et al. 2006; Nugnes et al. 2017; Triapitsyn 2015; Trjapitzin and Chiappini 1994; Viggiani and Nugnes 2018; Zanolli et al. 2016). However, an extensive recent review, considering both morphological characters and genetic analysis, suggested that the variability is intraspecific, rather than interspecific (Triapitsyn et al. 2020).

*Anagrus atomus* overwinters inside the eggs of alternative hosts on spontaneous vegetation surrounding vineyards (Boller et al. 2004; Ponti et al. 2003; Viggiani et al. 2006; Zanolli and Pavan 2011). In spring, the parasitoid moves into the vineyards where it completes several generations per year in *E. vitis* eggs (Cerutti et al. 1991). In autumn, *A. atomus* migrates back to the surrounding vegetation, where overwintering eggs are laid in eggs of leafhoppers other than *E. vitis* (Zanolli and Pavan 2011, 2013).

As with many other parasitoids, the mating system of *A. atomus* is characterized by protandry, with males emerging first followed by females (Zanolli and Pavan 2013). The sex ratio is generally 1:1 (Zanolli and Pavan 2011). *Anagrus atomus* females begin to lay eggs as soon as they emerge and have a life expectancy of 9 days at 24 °C (Agboka et al. 2004). In *A. atomus*, as in most hymenopteran parasitoids, male offspring emerge from unfertilized haploid eggs (arrhenotokous parthenogenesis) and female offspring from fertilized diploid eggs (Choudhury and Copland 2003; Heimpel and De Boer 2008).

In male parasitoids, the perception of the female sex pheromone is often followed by wing fanning (Böttinger et al. 2020; Vinson 1972). This behaviour has also been exploited for the purpose of pheromone isolation (Nazzi et al. 1995). When males of the closely related species *A. incarnatosimilis* Soyka and *A. breviphragma* Soyka recognize a virgin female, they raise their wings perpendicular to the dorsum and quickly move towards the female (Moratorio and Chiappini 1995). In *A. breviphragma*, mated females can still attract males such that, occasionally, a second insemination can occur (Moratorio and Chiappini 1995).

This study was carried out to ascertain the occurrence and identity of a female sex pheromone in the parasitoid *A. atomus* through behavioural bioassays and chemical analyses. The research included three consecutive steps: (1) verification of the existence of a female sex pheromone through behavioural bioassays; (2) identification of the chemical eliciting a response in males; (3) laboratory and field tests of the attractiveness of the identified compound towards males.

The information gathered on the female sex pheromone of *A. atomus* could be useful to monitor wasp populations in the field, to delineate their spatial distributions and to determine potential sources of parasitoids in the context of habitat management strategies.

## Methods and Materials

### Insect Rearing

*Anagrus atomus* was supplied by Biowise (Petworth, West Sussex, UK) as parasitized eggs of the leafhopper *Hauptidia maroccana* (Melichar) (Hemiptera: Cicadellidae) reared on *Primula* sp. plants. Parasitized eggs inside the leaf portions were individually isolated in vials (1.5 × 10 cm) and kept in a climatic chamber (Sanyo Versatile Environmental Test Chamber) at 60 ± 5% RH and 24 ± 1 °C with a daily light/dark cycle of 16:8 h. Honey was supplied to newly emerged adults used for bioassays. The vials were checked daily for adult emergence. Unmated males and females to be used in the bioassays were maintained in the same vial where development took place until use. Mated females were obtained by putting couples of newly emerged females and males in a vial until mating was observed and then placed back to their respective vial. After emergence parasitoid wasps were maintained under the same conditions as described above.

### Olfactometer Bioassays

All behavioural experiments were performed using a four-arms olfactometer (diameter 10 cm) (Pettersson 1970; Vet et al. 1983). Stimuli to be tested (i.e. live insects, extracts and pure substances on a strip of filter paper) were placed in a syringe connected with an arm upwind of the exposure chamber and closed at the opposite end with a mesh, so that one field of the olfactometer was treated with the odour to be tested and three others arms served as odourless control fields. The olfactometer was placed in a dark cardboard box to exclude the interference of possible external visual cues. All bioassays were carried out between 10:00 am and 6:00 pm at 24 ± 1.5 °C, in a darkened room. The olfactometer was illuminated from the top under a 7500 lx light source. The air stream was maintained at 400 ml/min. One hour before the beginning of an experimental session, parasitoid wasps in vials were placed in the test room to get acclimatized to experimental conditions. To avoid any asymmetrical bias, the olfactometer was rotated 90° clockwise after each bioassay. Parasitoid wasps were introduced individually through a hole in the centre of the olfactometer’s ceiling. The test started as soon as the insect entered the olfactometer and lasted for 10 mins. Each wasp was tested only once. The first choice made by the insects and the time spent in each odour field were recorded with the computer program “OLFA 1.0” (Nazzi 1995). After each test, the olfactometer was dismantled, scrupulously washed with 70% ethanol and then rinsed with water. If not otherwise specified in the different experiments, 12 parasitoid wasps per treatment were tested.

During the olfactometer assays, the male behaviour (i.e. general behaviour, wing fanning and locomotory activity) was also recorded.

### Behavioural Evidence of a Female Attractive Pheromone

To ascertain the existence of a female sex pheromone in *A. atomus*, four experiments were conducted. Experiments 1 and 2 were carried out to test the hypothesis that *A. atomus* females release a pheromone to attract males and whether male responsiveness is influenced by the age and mating status of the female. In Experiment 1 and 2, the response of males towards 0 to 4-day-old unmated females and towards 0 to 3-day-old mated females was evaluated, respectively. 0-day-old females were tested within 24 h from the emergence.

Experiment 3 was carried out to test the possible attraction of males to conspecific males, using 0 to 1-day-old unmated males both as bait and test insects.

Experiment 4 aimed at testing the response of males to the crude ether extract of 0 to 1-day-old unmated females. For this purpose, twelve 0 to 1-day-old unmated females were killed by freezing (30 mins, −20 °C) and then extracted with 120 μl of ether at room temperature for 15 mins. The resulting extracts were stored at −20 °C until use. For each of the 12 replicates of the bioassay, one female equivalent of the extract (10 μl) was applied to a strip of filter paper (absorbent paper 500 μm, 0.5 × 3.0 cm), and the solvent was allowed to evaporate. Then the strip was inserted into the syringe connected to the treated arm of the four-arm olfactometer. For each replicate a new strip of paper was used.

### Identification of a Candidate Pheromone

To isolate and identify the female sex pheromone demonstrated as above, we carried out Experiment 5. This involved testing the biological activity of two fractions of the female extract using 0 to 1-day-old unmated males as test insects. Both males and females were collected and frozen within 24 h after emergence without feeding them. Ether extracts of *A. atomus* females and males were fractionated by liquid chromatography, and the resulting fractions bioassayed as usual. Batches of 15 freshly emerged females were extracted with 150 μl of ether at room temperature as described above, then the extract was loaded on a column packed with 100 mg silica gel (200–400 mesh, pore size 60 Å. Sigma Aldrich US) and eluted sequentially with 1 ml each of hexane and ether. One female equivalent of each fraction in 10 μl of the solvent was tested in the olfactometer against 16 individual *A. atomus* males. Ether and hexane alone were also tested.

### Chemical Analysis of the Crude Extract

Ether extracts of males and females (*N* = 10 for each sex) were prepared as described above and analysed by coupled gas chromatography-mass spectrometry (GC-MS) on a Varian 3400 gas chromatograph coupled with a Varian Saturn 2000 mass spectrum detector, equipped with CIP-SIL 8 capillary column (30 m × 0.25 mm I.D.; 0.25 μm thickness). The oven temperature was 50 °C for 1 min, followed by a ramp to 320 °C at 10 °C/min; final temperature was held for 2 mins. The carrier gas was He maintained at a constant flow rate of 1 ml/min. Injected volume was 1 μl (splitless) and the injection temperature was 250 °C. The interface temperature was maintained at 250 °C. Mass Spectrometry (MS) detection was performed with electron impact (EI) mode at 70 eV by operating in the fil-mul delay in the 40–650 amu range. The identification of the volatile compounds emitted by females but not by males was performed by comparison of crude extracts from males and females. Unknown spectra were identified using the NIST Library (National Institute of Standards and Technologies, US) as a reference for spectra and retention indexes.

### Coinjection of the Female Crude Extract and Synthetic Eugenol

An ether extract of females (*N* = 50) was prepared as described above, reduced under nitrogen to 5 μl, and 2 μl of this extract analysed by GC-MS. Then 1 μl of a solution of synthetic eugenol in ether (0.01 μg/μl, Sigma Aldrich US) was added to the remaining extract and 1 μl of this mixture analysed by GC-MS. This analysis also served for the purpose of roughly quantifying the amount of eugenol associated with the major peak found in the female extract but not in the male one.

### Testing of the Candidate Sex Pheromone Component

In Experiment 6, the activity of synthetic eugenol on males and females was evaluated in olfactometer bioassays. Responses of *A. atomus* males (*N* = 16) to eugenol were assessed at different doses (0.1, 0.5, 1, 5, 10 and 100 ng). Ether was used as a control as the treatment stimulus was dissolved in that solvent.

The response to eugenol was also tested with 16 individual *A. atomus* unmated females (*N* = 16) in order to exclude the possibility that the substance is an aggregation rather than a sex pheromone. Ether was used as control as above.

### Cage Bioassays

To confirm the attractiveness of eugenol in the laboratory, the response of 0 to 1-day-old unmated males was tested in a Plexiglas’s cage with ventilation holes covered with mesh (30 × 60 × 50 cm) and exposed to natural light. Fifty μl of an eugenol solution corresponding to 50 ng of pure compound were pipetted onto a strip of filter paper (absorbent paper, 0.5 × 3.0 cm) and, after evaporation of the solvent, the strip was fixed in the middle part of a yellow sticky trap (15 × 5 cm). Another trap baited with 50 μl of ether was used as a control. Traps were hanging from the top of the cage at a distance of 40 cm from each other and males were released inside once a day. The bioassay lasted 5 days. Every 24 h, a batch of five males was released and the traps were replaced. The number of males captured on traps was counted.

### Field Experiments

This study was carried out in an insecticide unsprayed vineyard (cultivar Tocai Friulano) located in a hilly grape-growing area (locality Buttrio, 46° 01′ latitude N, 13° 21′ longitude E, 90 m a.s.l.). Yellow sticky traps (11.5 × 24 cm) smeared with Temoocid® (Kollant S.r.l., Vigonovo, Venice, Italy) were used for *A. atomus* monitoring (Picotti and Pavan 1993; Zanolli and Pavan 2011). Six traps baited with eugenol were compared with six control traps. Lures were prepared with 9 μg of synthetic eugenol in glass micro-vials (1 × 3 cm) with perforated plastic caps. Under laboratory conditions vials used as bait released about 20 ng of eugenol per day, but under field conditions greater release is likely. On each baited trap a vial with eugenol was hanged in the upper part, while in the control traps empty vials were applied. Traps were distributed in the vineyard following a randomized blocks scheme with six replicates (rows). The distance between the control and the treated traps was at least 20 m both along the rows and between the contiguous rows. Traps were replaced twice a week and baits were changed at each replacement. *Anagrus atomus* males captured at each sampling interval were counted in the laboratory under a dissection microscope. Within the *A. atomus* species, the intraspecific taxa *A. atomus* and *A. parvus* Soyka were also distinguished (Zanolli et al. 2016).

### Statistical Analysis

Wasp residence time in the treated arm was compared to the average time spent in the control arms of the olfactometer using a paired one-tailed *t-*test. This test was adopted because: i) residence time in the olfactometer arms appeared to be normally distributed according to Kolmogorov and Smirnov method; ii) no significant differences among the control arms were detected using ANOVA; iii) since the olfactometer was rotated every minute, residence time in control arms could be considered as belonging to the same population of data; iv) a paired test appeared to be important to account for the high inter-individual variability of test insects, since different wasps were used in each replicate of each bioassay; v) the same statistical approach was adopted in similar studies (e.g. Drakulic et al. 2015; Fancelli et al. 2018; Mao-xin et al. 2008).

To compare the male response to females of different ages or physiological status (unmated/mated), the proportions of residence time in the treated arm with respect to control arms were calculated and before statistical analysis data were arcsine transformed. To compare the male response to females of different age, ANOVA and *Tukey* tests were performed, whereas to compare the male response to unmated/mated females an unpaired *t-*test was performed.

Number of males captured on yellow sticky traps in cage bioassays was compared with a Binomial test.

To compare the total field captures of *A. atomus* males on the eugenol baited and control traps along the monitoring periods, the *Wilcoxon* matched-pairs signed-ranks test was applied considering sampling dates as replicates.

Statistical analyses were performed with GraphPad Instat 3.0 for Macintosh.

## Results

### Behavioural Evidence of a Female Produced Sex Pheromone

**Fig. 1:**
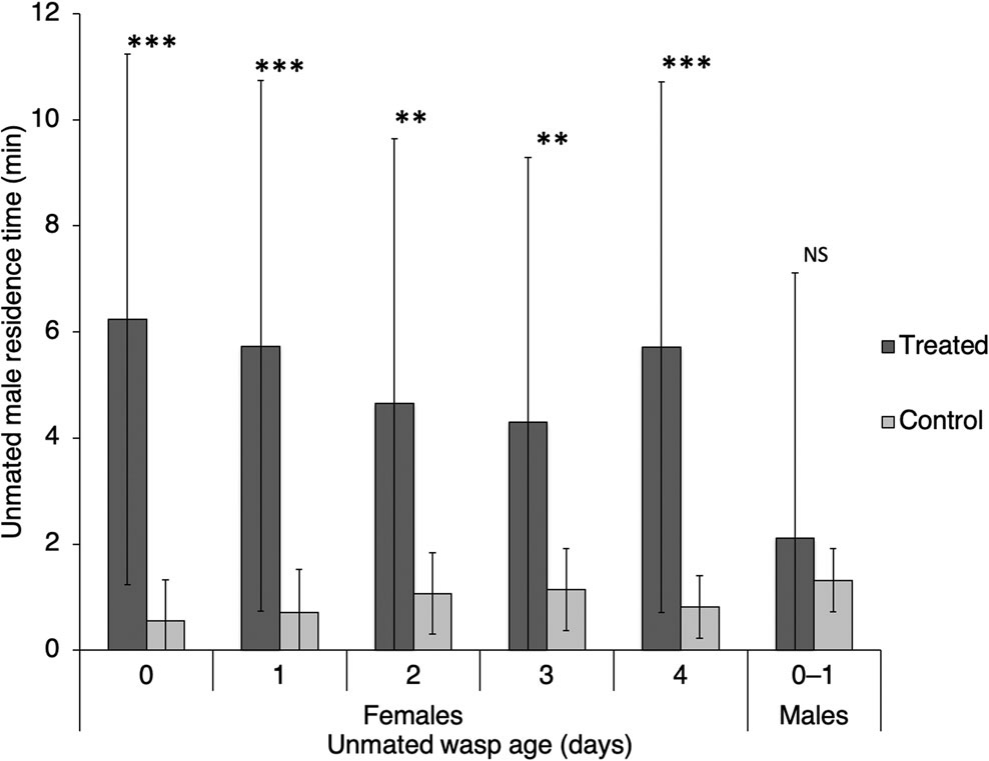
Time spent by *Anagrus atomus* males (± standard deviation) in the arms of an olfactometer treated or not with the odour of one unmated *A. atomus* female of different ages or one-day-old *A. atmous* males. Asterisks mark statistically significant differences (** = *P* < 0.01; *** = *P* < 0.001; NS = no significant differences)

In Experiment 1 males were significantly attracted towards the olfactometer arm containing the odour of a virgin female (paired *t* test*, N* = 12; 0 days: *t* = 6.21, *P* < 0.001; 1 day: *t* = 7.12, *P* < 0.001; 2 days: *t* = 4.0, *P* = 0.002; 3 days: *t* = 3.86, *P* = 0.003; 4 days: *t* = 6.85, *P* < 0.001; Fig. 1). Attraction was not significantly affected by female age (unpaired ANOVA, df = 4, *F* = 0.87, *P* = 0.49).

**Fig. 2:**
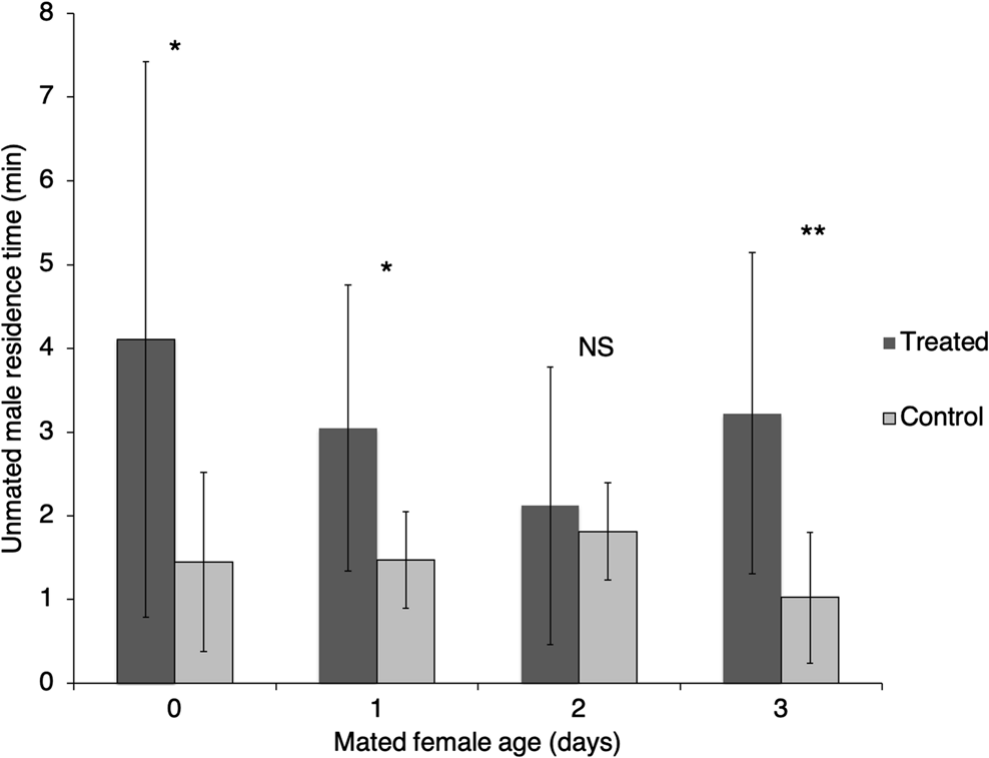
Time spent by *Anagrus atomus* males (± standard deviation) in the arms of an olfactometer treated or not with the odour of a mated female of different ages. Asterisks mark statistically significant differences (* = *P* < 0.05; ** = *P* < 0.01; NS = no significant differences)

In Experiment 2, mated females also elicited a positive male response (Fig. 2). No significant attraction was found on day 2 after female mating, but significance resumed at day 3 (paired *t* test*,* *N* = 12; 0 days: *t* = 2.13, *P* = 0.028; 1 day: *t* = 2.60, *P* = 0.012; 2 days: *t* = 0.53, *P* = 0.30; 3 days: *t* = 2.70, *P* = 0.002). Overall, female age did not seem to affect male response (unpaired ANOVA, df = 3, *F* = 1.69, *P* = 0.18).

Moreover, in both Experiments 1 and 2, males exposed to the odour released by a virgin female showed wing fanning and increased locomotory activity. In particular, most males fan their wings in the presence of a virgin female, whereas only a small proportion of those exposed to the odour of a mated female did so.

**Fig. 3:**
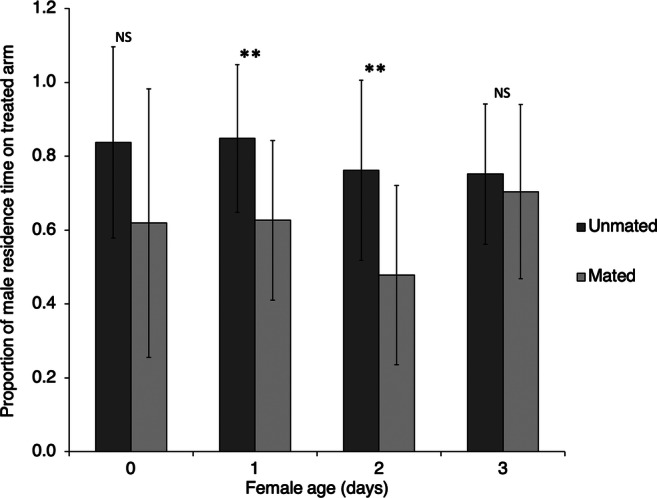
Proportion of the residence time of *Anagrus atomus* males in the arm of the olfactometer treated with the odour of females of different physiological status and age. Asterisks mark statistically significant differences (** = *P* < 0.01; NS = no significant differences)

The comparison between the proportion of time that males spent in the arm of the olfactometer treated with the odour of a virgin or a mated female revealed that 1- and 2-day-old males were significantly more attracted to unmated females than to mated ones (unpaired *t* test*,* *N* = 12; 0 days: *t* = 2.04, *P* = 0.054, 1 day: *t* = 3.10, *P* = 0.005; 2 days: *t* = 3.04, *P* = 0.006; 3 days: *t* = 0.47, *P* = 0.64; Fig. 3).

In Experiment 3, males did not stay longer in the arm of the olfactometer treated with the odour of a young male (paired *t* test, *N* = 12, *t* = 1.49, *P* = 0.08; Fig. 1).

**Fig. 4:**
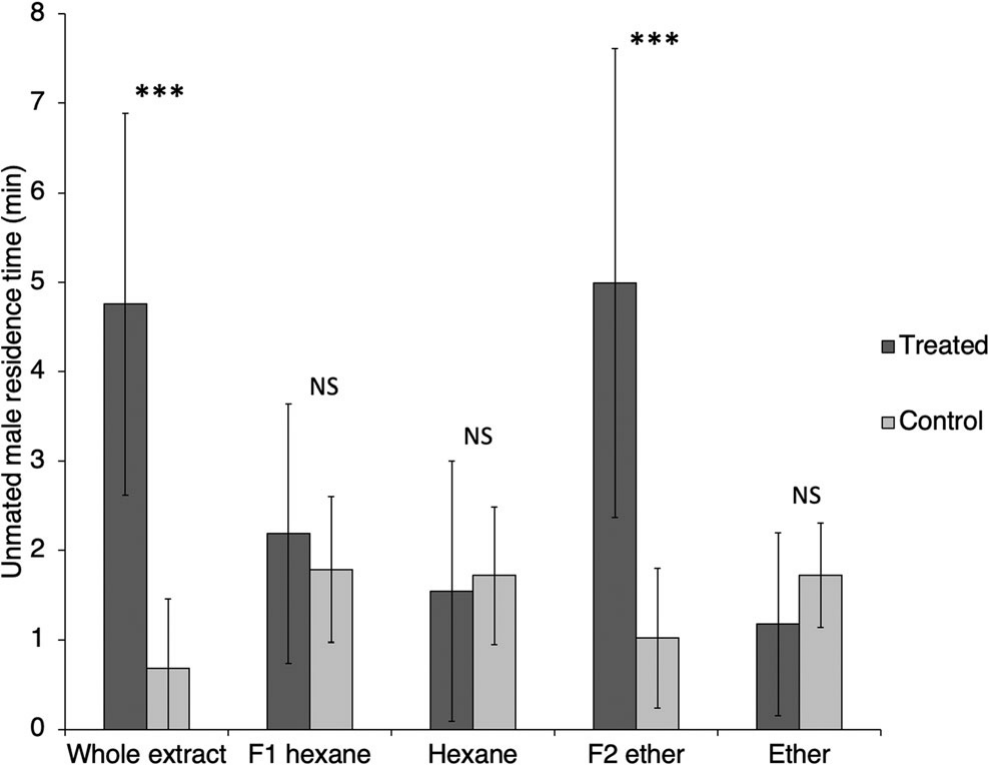
Time spent by *Anagrus atomus* males (± standard deviation) in the treated and control arms of an olfactometer. The stimuli used for treating one arm of the olfactometer are the whole extract of *A. atomus* unmated females, the apolar and polar fractions of *A. atomus* unmated females extracts (F1 hexane and F2 ether, respectively) and the two solvents alone (hexane and ether). Asterisks mark statistically significant differences (*** = *P* < 0.001; NS = no significant differences)

In Experiment 4, males showed a strong attraction towards a one-female-equivalent of the crude extract of unmated females (paired *t* test, *N* = 16, *t* = 7.43, *P* < 0.001; Fig. 4). The male response to the extract was similar to that displayed to a living unmated female, although no wing fanning was noted in this case.

### Identification of a Candidate Pheromone

In Experiment 5, the more polar ether fraction of a female extract elicited a significant behavioural response from males (paired *t* test, *N* = 12, *t* = 5.05, *P* < 0.001), whereas neither the apolar fraction (paired *t* test, *N* = 12, *t* = 0.66, *P* = 0.26) nor ether (paired *t* test, *N* = 7, *t* = 1.43, *P* = 0.09) or hexane (paired *t* test, *N* = 12, *t* = 0.34, *P* = 0.37), used to extract the polar and the apolar hexane fractions respectively, significantly affected the male behaviour (Fig. 4).

### Chemical Analysis of *A. atomus* Crude Whole Extract

**Fig. 5:**
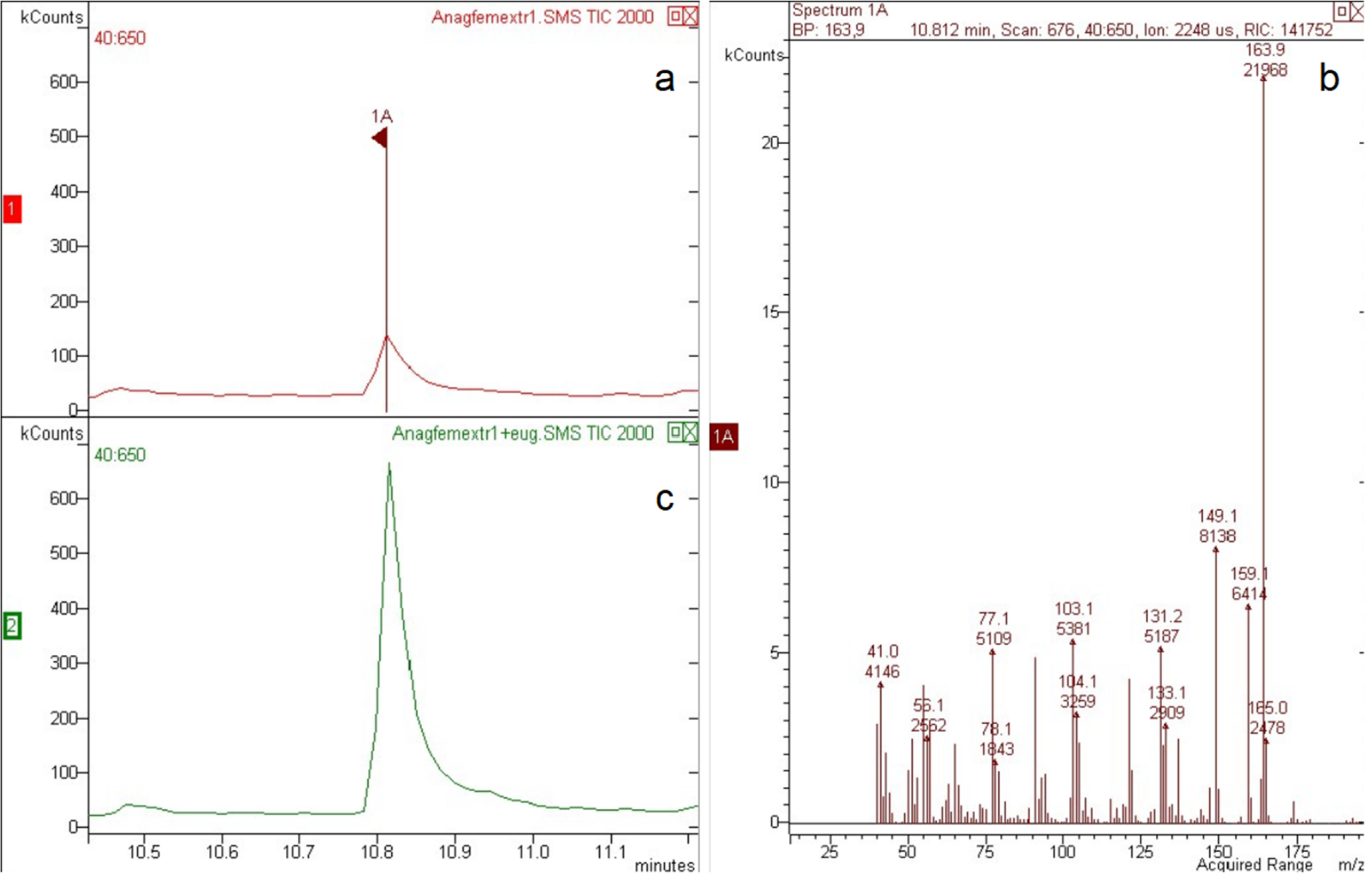
Chromatogram of the crude extract of 50 virgin *Anagrus atomus* females (**a**). The single highlighted peak, found only in the females’ extract, was identified as eugenol (the corresponding spectrum is reported in panel **b**) and confirmed by coinjection of standard eugenol (**c**)

The comparison of GC-MS analyses of virgin males’ and females’ crude extracts revealed only minor differences in the quantity of a few identified hydrocarbons. The most notable difference was one peak that was found only in the female extracts (Fig. 5). The peak was identified as eugenol by comparison of the mass-spectrum and retention time with of an authentic standard and confirmed by coinjection. The calculated amount of eugenol per female was less than 1 ng.

### Testing of the Candidate Pheromone

**Fig. 6:**
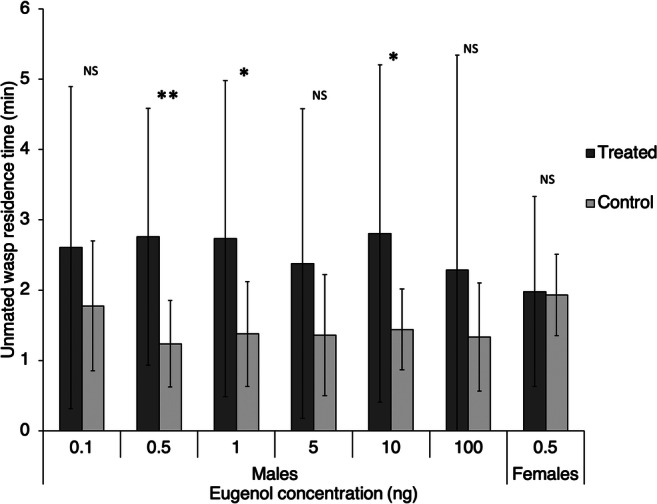
Time spent by *Anagrus atomus* males and females (± standard deviation) in the arms of an olfactometer treated or not with different doses of synthetic eugenol. Asterisks mark statistically significant differences (* = *P* < 0.05; ** = *P* < 0.01; NS = no significant differences)

In Experiment 6 male wasps were attracted to synthetic eugenol in the olfactometer responding to several doses ranging from 0.5 ng to 10 ng (paired *t* test, *N* = 16, 0.1 ng: *t* = 1.07, *P* = 0.15; 0.5 ng: *t* = 2.71, *P* = 0.008; 1 ng: *t* = 1.98, *P* = 0.033; 5 ng: *t* = 1.46, *P* = 0.08; 10 ng: *t* = 1.92, *P* = 0.037; 100 ng: *t* = 1.05, *P* = 0.16; Fig. 6). Unmated females did not show attraction towards the dose that appeared to be most attractive to males (paired *t* test*,* *N* = 16, *t* = 0.10, *P* = 0.46; Fig. 6).

### Cage Bioassays

**Fig. 7:**
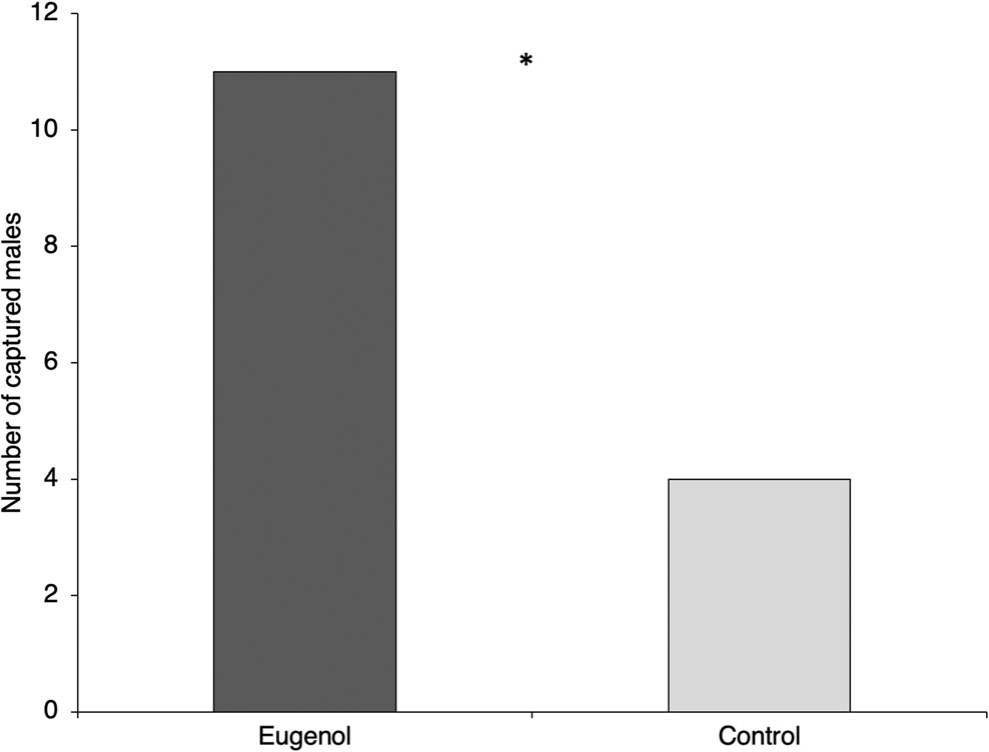
*Anagrus atomus* males captured per day on yellow sticky traps baited or not (control) with 50 ng of synthetic eugenol and maintained in a small box under artificial conditions (* = *P* < 0.05)

Under lab conditions yellow sticky traps baited with eugenol captured more caged males than control traps (binomial test, *P* = 0.02; Fig. 7).

### Field Experiments

**Fig. 8:**
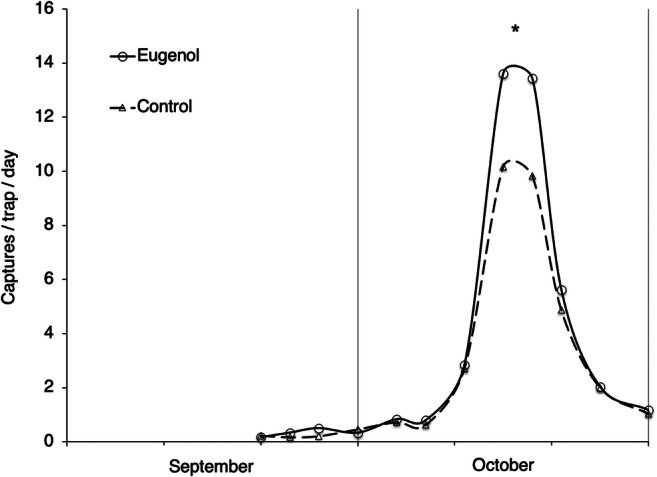
*Anagrus atomus* males captured in a vineyard on yellow sticky traps baited or not with eugenol (* = *P* < 0.05)

Under open field conditions yellow sticky traps baited with eugenol captured more *A. atomus* males than control traps (*Wilcoxon* matched-pairs signed-ranks test, *W* = 16.5, *N* = 13, *P* < 0.05; Fig. 8). If only the intraspecific taxa *A. atomus* was considered within *A. atomus* species, the differences were also significant (*Wilcoxon* matched-pairs signed-ranks test, *W* = 18.0, *N* = 13, *P* < 0.05).

## Discussion

The attraction of *A. atomus* male wasps to both live virgin females and their crude extracts supports the hypothesis that a volatile sex pheromone is released by females, similar to what has previously been found in other hymenopteran parasitoids (De Lury et al. 1999; Eller et al. 1984; Nazzi et al. 1995; Ruther et al. 2000; Salerno et al. 2012; Simser and Coppel 1980). Attraction of males, but not females, confirms that this is not an aggregation pheromone.

Olfactometer and cage bioassays suggest that the attractant is a long-range pheromone, since the distance at which it is perceived by *A. atomus* males is greater than 2 cm (Keeling et al. 2004). The females of other *Anagrus* species, such as *A. delicatus* (Cronin and Strong 1990), *A. incarnatosimilis* and *A. breviphragma* (Moratorio and Chiappini 1995), mate at the emergence site. For this reason, it is likely they do not produce a long-range attractant analogous to other hymenopteran parasitoids showing the same reproductive behaviour (Godfray 1994). In fact, in this latter case, males emerge first and then wait for the female’s emergence to mate on site. In contrast, *A. atomus* does not mate at the emergence site because it parasitizes solitary eggs (Agboka et al. 2004), and, since only males are normally produced when females do not meet a male before egg laying, a female produced long range attractant facilitates male search. Natural selection may favour sex pheromone production if the population is not at sex ratio equilibrium or if there are advantages in having the capability of producing both sons and daughters (Godfray 1994). Metabolic costs are involved in the production of long-range sex pheromones and so it is likely that *A. atomus* females gain an advantage from mating.

Although some differences in male response to unmated and mated females were noted, they both appeared to be attractive to males. Therefore, the production/release of the pheromone by *A. atomus* females seems not to cease after mating, as would be expected if multiple matings occurred. Polyandry is known in other mymarids such as, for example *Anaphes nitens* (Santolamazza-Carbone and Pestaña 2010) and a second mating was occasionally observed also in *A. breviphragma* (Moratorio and Chiappini 1995). The fitness returns from multiple matings of old females are not clear since egg supply and quality usually decrease with mother age (Giron and Casas 2003). Nevertheless, a high tendency of old females to re-mate was observed in other wasps such as *Nasonia vitripennis* (Burton-Chellew et al. 2007) and could be related to the limited storage capacity of the spermatheca (Santolamazza-Carbone and Pestaña 2010). In haplodiploid hymenopteran species, sperm depletion followed by the oviposition of unfertilized male eggs is possible (Godfray 1990). In this case, since the evolutionary stable strategy sex ratio in panmictic populations is 1:1, as reported for *A. atomus* in previous studies (Zanolli and Pavan 2011), sperm-depleted females are likely to re-mate thus explaining the persistence of a female produced attractant after mating. The release of sex pheromone by mated females could be convenient if multiple matings increase lifespan of females, as observed in *Trichogramma evanescens* (Jacob and Boivin 2005), or if sex pheromones have multiple functions, as in *Venturia canescens* (Metzger et al. 2010).

The compound triggering male attraction was identified as eugenol (4-allyl-2-methoxyphenol), a sex pheromone component previously detected in male butterflies of the genus *Amauris* spp. (Lepidoptera, Danaidae) (Schulz et al. 1993) and in *Bactrocera* spp. (Symonds et al. 2009). No more compounds were identified in the active fraction of the crude extract, but the limited quantity of material extractable from females may have precluded the identification of other compounds. In fact, the magnitude of the biological effect elicited by several doses of the compound appeared to be smaller than that observed against live females. The presence of eugenol only in the female extract was confirmed also by analysing males and females of *A. atomus* emerged from grapevine leaves collected in the field (data not shown).

Furthermore, wing fanning has never been observed when extracts or pure compounds were tested in the bioassay. Since many hymenopteran parasitoid females produce sex pheromones that are constituted of a blend of two or more compounds, one attracting the males and others mediating the subsequent phases of the courtship behaviour (Quicke 1997; Ruther 2013), the hypothesis that more compounds involved in *A. atomus* reproduction are still to be identified is very likely. Nevertheless, this is the first study in which a sex-pheromone component was identified in Mymaridae.

Under open field conditions, eugenol significantly increased the already elevated captures of *A. atomus* males in yellow sticky traps, confirming the biological activity detected under laboratory conditions. Unbaited yellow sticky traps have already proved to be a valid tool to monitor field populations of *A. atomus* wasps (Picotti and Pavan 1993; Zanolli and Pavan 2011). A complete identification of the pheromone blend of this species could significantly increase the efficiency of such traps and their potential for monitoring of this beneficial insect as a bioindicator of the effects of chemical control and cultural practices on natural enemies (Gabrýs et al. 1997; Suckling et al. 2002).
